# A global search for novel transcription factors impacting the *Neurospora crassa* circadian clock

**DOI:** 10.1093/g3journal/jkab100

**Published:** 2021-04-19

**Authors:** Felipe Muñoz-Guzmán, Valeria Caballero, Luis F Larrondo

**Affiliations:** ANID—Millennium Science Initiative Program—Millennium Institute for Integrative Biology (iBio), Santiago 8331150, Chile; Departamento de Genética Molecular y Microbiología, Facultad de Ciencias Biológicas, Pontificia Universidad Católica de Chile, Santiago 8331150, Chile; Departamento de Genética Molecular y Microbiología, Facultad de Ciencias Biológicas, Pontificia Universidad Católica de Chile, Santiago 8331150, Chile; ANID—Millennium Science Initiative Program—Millennium Institute for Integrative Biology (iBio), Santiago 8331150, Chile; Departamento de Genética Molecular y Microbiología, Facultad de Ciencias Biológicas, Pontificia Universidad Católica de Chile, Santiago 8331150, Chile

**Keywords:** circadian clock, *Neurospora crassa*, transcription factors, luciferase, reverse genetic screen

## Abstract

Eukaryotic circadian oscillators share a common circuit architecture, a negative feedback loop in which a positive element activates the transcription of a negative one that then represses the action of the former, inhibiting its own expression. While studies in mammals and insects have revealed additional transcriptional inputs modulating the expression of core clock components, this has been less characterized in the model *Neurospora crassa*, where the participation of other transcriptional components impacting circadian clock dynamics remains rather unexplored. Thus, we sought to identify additional transcriptional regulators modulating the *N. crassa* clock, following a reverse genetic screen based on luminescent circadian reporters and a collection of transcription factors (TFs) knockouts, successfully covering close to 60% of them. Besides the canonical core clock components WC-1 and -2, none of the tested transcriptional regulators proved to be essential for rhythmicity. Nevertheless, we identified a set of 23 TFs that when absent lead to discrete, but significant, changes in circadian period. While the current level of analysis does not provide mechanistic information about how these new players modulate circadian parameters, the results of this screen reveal that an important number of light and clock-regulated TFs, involved in a plethora of processes, are capable of modulating the clockworks. This partial reverse genetic clock screen also exemplifies how the *N. crassa* knockout collection continues to serve as an expedite platform to address broad biological questions.

## Teaser

In order to identify new players modulating the circadian clock in the model Neurospora crassa, we adopted a reverse genetics strategy. Thus, we focused on transcription factors knockouts and crossed them to strains containing circadian luciferase reporters. Our screen covered close to 60% de the 302 genes encoding for such proteins in Neurospora, identifying that 23 of them appear to modulate period, while none of the tested ones (besides the classic core-clock components) are essential for daily rhythms.

## Introduction

Circadian rhythms are a widespread phenomenon across the tree of life, conferring individuals the capacity to coordinate cellular and organismal metabolism, physiology, and behavior to day/night cycles (Dunlap 1999; Loudon 2012; Montenegro-Montero and Larrondo 2016; Dunlap and Loros 2017). These rhythms exhibit periodic oscillations *circa* 24 hours, which can be sustained even in the absence of environmental signals, and can be synchronized to external cues such as light and temperature (Lakin-Thomas and Brody 2004; Narasimamurthy and Virshup 2017; Kuhlman *et al.* 2018). The circadian core oscillator is composed of a topologically conserved transcriptional-translational negative feedback loop (TTFL), although the molecular composition of the core components differs across phyla (Merrow *et al.* 2010; Zhang and Kay 2010; Zheng and Sehgal 2012). In the model fungus *Neurospora crassa*, the positive element is the white collar complex (WCC), a heterodimer formed by the TFs white collar 1 (WC-1) and white collar 2 (WC-2). The WCC is responsible for activating transcription of the *frequency* gene, which encodes for the negative element (FRQ) that feeds back to inhibit the activity of WCC, therefore closing the loop. As FRQ is progressively phosphorylated by several kinases, including CK1, its affinity for the latter as well as for WCC diminishes, therefore no longer inactivating its own expression. As this occurs, hyperphosphorylated FRQ is subjected to proteasomal degradation, all of which can be visualized as daily oscillations in *frq* mRNA and protein levels (Dunlap 1999; Montenegro-Montero *et al.* 2015; Diernfellner and Brunner 2020). The changing levels and activities of these two core components allow the entire system to oscillate (Aronson *et al.* 1994a,b; Brunner and Kaldi 2008; Neiss *et al.* 2008). However, different aspects of how eukaryotic clocks keep their period constant, and are robust to external perturbations, remain partially unsolved (Ripperger and Brown 2010; Brown *et al.* 2012; Partch *et al.* 2014; Kramer 2015; Hurley *et al.* 2016).

Mechanisms involved in eukaryotic clock regulation comprise several layers of modulation, such as chromatin remodeling, transcriptional control, alternative splicing, antisense transcripts, post-transcriptional regulation and, a high degree of post-translational modifications (phosphorylation) of clock proteins (Brunner and Schafmeier 2006; Ripperger and Brown 2010; Kojima *et al.* 2011; Hurley *et al.* 2014, 2016; Montenegro-Montero *et al.* 2015; Mendoza-Viveros *et al.* 2017). In plant and animal models, transcriptional regulation of clock components had been profusely addressed, leading to the identification of several transcription factors (TFs) which participate in accessory transcriptional loops interlocked with the central core oscillator (Honma *et al.* 2002; Kadener *et al.* 2007; Rossner *et al.* 2008; Ripperger and Brown 2010; Zhang and Kay 2010; Hardin 2011; Lowrey and Takahashi 2011; Nagel and Kay 2012; Zheng and Sehgal 2012; Ronald and Davis 2017). In *N. crassa*, the WCC plays a pivotal role in mastering *frq* expression, although other transcriptional regulators have been found to fine-tune it such as IEC-1 (Gai *et al.* 2017) and CBF-1 (Cao *et al.* 2018), or indirectly do so by modulating WCC abundance upon changes in sugar levels, impacting metabolic compensation, as observed for CSP-1 and RCO-1 (Sancar *et al.* 2012; Olivares-Yanez *et al.* 2016). Thus, these regulators are capable of affecting the expression of core clock components, having an effect on key circadian parameters (Sancar *et al.* 2011, 2012; Olivares-Yanez *et al.* 2016; Gai *et al.* 2017). Nevertheless, besides this limited list of transcriptional modulators, there is scarce information regarding additional TFs impacting clock function in *N. crassa*, in contrast to what has been elucidated in other models. (Zhang and Kay 2010; Zheng and Sehgal 2012). Indeed, while there is evidence that in *N. crassa* the core TTFL is interlocked with a positive feedback loop in which FRQ posttranslationally upregulates WC-1 levels by stabilizing it, there is no information on which TFs may be also contributing to ancillary positive feedback loops that modulate WCC components. Thus, although there is a slight positive direct effect of WCC over *wc-1* transcription (Kaldi *et al.* 2006; Sancar *et al.* 2012), FRQ favors *wc-2* expression through an indirect and still unknown mechanism, involving unidentified TFs (Cheng *et al.* 2001; Neiss *et al.* 2008).

The ease in conducting genetic analyses (Beadle and Tatum 1941) and the robust circadian phenotype of daily spore production (banding) (Sargent *et al.* 1966; Montenegro-Montero *et al.* 2015), have made *N. crassa* a key model for unveiling the molecular details of clocks (Dunlap 1993, 2008; Brody 2011). This has been aided by forward genetics analyses of naturally occurring or induced mutations (Feldman and Hoyle 1973; Feldman and Atkinson 1978). However, these banding-based screens can yield confounding results by identifying mutations that affect conidiation *per se* and not necessarily the core clock (He *et al.* 2003; Zhou *et al.* 2018). Nevertheless, in recent years it has been possible to conduct bioluminescence-based studies, utilizing luciferase as a proxy to follow circadian gene expression reporting, unambiguously, the status of the core clock (Gooch *et al.* 2008; Larrondo *et al.* 2012; Montenegro-Montero *et al.* 2015). Importantly, luciferase clock reporters can reveal normal clock function in strains that otherwise may appear arrhythmic, or devoid of circadian banding in race tube assays (He *et al.* 2003; Shi *et al.* 2007; Cha *et al.* 2015; Larrondo *et al.* 2015; Montenegro-Montero *et al.* 2015; Olivares-Yanez *et al.* 2016). Taking all these into account, we adopted a reverse genetic approach, utilizing the *N. crassa* Knockout (KO) collection (Colot *et al.* 2006) as well as luminescent reporters to analyze circadian phenotypes in different genetic backgrounds. Herein, we describe such efforts focused on TFs, aiming at identifying the ones that when absent modulate clock period. Future work will provide further understanding on how these TFs regulate circadian properties either by modulating core clock components expression, or by other mechanisms.

## Materials and methods

### Strains and crosses

For this reverse genetic screen, we analyzed the sexual progeny obtained from crosses between circadian luciferase reporter strains and TF KOs available from the Fungal Genetic Stock Center (FGSC, Kansas City, MO, USA). The KOs were obtained as part of the *N. crassa* Functional Genomics Project, where individual loci were replaced with the *hygromycin B phosphotransferase* (*hph*) gene drug-resistance cassette (Colot *et al.* 2006; Collopy *et al.* 2010). We generated a list of putative TF-encoding genes based on the presence of the DNA binding domain sequence described for the *N. crassa* genome available in the web platform CISBP (cisbp.ccbr.utoronto.ca) (Weirauch *et al.* 2014). Such list included other genes previously related to transcriptional function (Borkovich *et al.* 2004; Tian *et al.* 2011), and which has been recently compiled (Carrillo *et al.* 2017), yielding a final list of 302 putative TFs loci listed in the Supplementary Table S1.

The reporter utilized in the primary screen was a firefly luciferase gene under the control of a minimal *frq* clock promoter (*frq_c-box_-luc*) integrated into the *his-3* locus in LG I: *his-3::frq_c-box_-luc* (Gooch *et al.* 2008; Larrondo *et al.* 2015). Alternatively, another reporter consisting on a destabilized firefly luciferase (containing a PEST domain), under the control of the same minimal *frq_c-box_* promoter was utilized, this time targeted to the *csr-1* locus: *csr-1::frq_c-box_-luc^PEST^*, as previously described (Cha et al. 2015; Olivares-Yanez *et al.* 2016). Both loci are ∼3 million bp apart, improving the number of successful crosses to that linkage group, by diminishing cosegregation of the reporter and hygromycin cassettes (Bardiya and Shiu 2007; Gooch *et al.* 2008; Honda and Selker 2009; Gooch *et al.* 2014). To confirm that the observed effects were not due to some spurious factor arising during the cross, and to also test the extent of circadian alterations caused by the missing gene, we also conducted a secondary screen utilizing a circadian output reporter. For this, we used *con-10^lu^*^c^ (*con-10^luc-bar^*) (Lauter and Yanofsky 1993; Olivares-Yanez *et al.* 2016), which corresponds to a fusion of luciferase to the *con-10* ORF at its endogenous locus. And while technically *con-10^luc^* is a translational reporter, it faithfully recapitulates core circadian alterations as *frq_c-box_-luc* does (Olivares-Yanez 2015) and, therefore, it helps further characterizing the mutants of interest. The reporters were introduced in lab strains derived from crosses between progenies of *87-74* and FGSC #9568 (Larrondo *et al.* 2015; Olivares-Yanez 2015) which were used as the parental strains for the crosses. They were also utilized as WT controls, along with selected hygromycin sensitive siblings from the progenies derived from the screen crosses.

Out of a list of 302 putative TFs encoding genes in the *N. crassa* genome (Montenegro-Montero 2014), 45 KO strains were not available in the *N. crassa* FGSC KO collection (http://www.fgsc.net/) at the time we obtained the arrayed strains from the FGSC.

### Growth conditions

Culture conditions for vegetative growth and asexual reproduction utilized Vogel minimal medium (VM) (Vogel 1956), whereas conditions for sexual development used synthetic crossing medium (SCM) (Westergaard and Mitchell 1947). Sorbose-containing medium (FIGS) was used for colony isolation on plates and ascospore germination and isolation (Davis and de Serres 1970). Picked ascospores were then grown on slants containing VM media supplemented with hygromycin (200 μg/ml; Calbiochem, San Diego, CA, USA) and luciferin (GoldBio) (10 μM), in order to select for progenies carrying knockout cassettes and reporter activity, respectively.

To conduct the circadian analyses (see below), spores from the selected progenies were inoculated in a 96 well plate containing LNN-CCD media (Larrondo *et al.* 2015; Olivares-Yanez 2015), with 25 μM of Luciferin (GoldBio). Cultures were grown for 24 hours in constant light (LL) at 25°C and then were analyzed under free-running conditions; consisting of constant darkness (DD) and 25°C, for 4–5 days in Percival incubators equipped with CCD PIXIS 1024B cameras (Princeton Instruments). As part of the high-throughput design, several 96 well-plates were run together in a single CCD camera run.

### Luciferase data analysis and statistical tests

The resulting images series obtained from CCD camera runs were analyzed with a customized script for ImageJ (Larrondo *et al.* 2012). The acquired data sets varied in some cases containing 2 or 3 pictures per hour, with exposition times of 10 or 5 minutes respectively, a difference that does not affect the analyses as information can be compared throughout the data sets. Importantly, control wild-type (WT) strains were included in each 96-well plate and each experimental run. The obtained luciferase traces were analyzed as raw as well as normalized data sets (see below).

For the circadian analysis of the recorded time series, the data were uploaded as individual CCD camera runs (each run containing the corresponding WT controls) in BioDare2 (***Bio***logical ***Da***ta ***Re***pository ***2***), a free-available online platform (https://biodare2.ed.ac.uk/) (Moore *et al.* 2014; Zielinski *et al.* 2014) which provides a comprehensive analysis of circadian parameters using different algorithms (Zielinski *et al.* 2014). The data were processed in the following manner for the primary screen: first, data were detrended to remove stationary effects over the time series, as such trends can cause distortions in the data masking circadian information. In addition, we discarded the first and last 12 hours of the data to minimize noise effects associated with the transition from light to darkness and improve subsequent detrending. Periods were calculated using a fast Fourier transform-nonlinear least-squares analysis (FFT-NLLS). Finally, we normalized and aligned every data point respect to the average of all points in their time series to facilitate their visualization (Plautz *et al.* 1997; Zielinski *et al.* 2014). The entire data sets produced in this genetic screen are stored in the BioDare2 platform, as an open access repository, with the spirit of propelling further analysis of these and other data by the circadian community. Importantly, the data sets from each CCD camera run are enlisted in the platform as “*Neurospora TF Circadian Clock*” plus the date of the CCD camera run; these entries also describe the experimental conditions, reporters and the TF KOs analyzed in each run. Period was re-calculated (see below) to compare this circadian parameter for each strain with an internal control (WT) in each 96-experimental run, to minimize the potential noise when comparing different plates in different experiments. The WT strains used in each plate and camera run were the parental reporters used for the crosses, their siblings, along WT siblings (hygromycin sensitive) of the KO crosses. The averaged period calculated for these WT is 21.76 hours, similar to the previous reported value of ∼21.5 utilizing similar WT controls (Larrondo *et al.* 2015). For the analyses, we calculated period change (Δτ; as Period_*KO—*_Period_*wt*_.), measured in hours.

We defined the tolerance interval for the WT population using their Δτ in each of the experiments, taking three standard deviation from the mean population (Zhang *et al.* 2009), after confirming their normal distribution (Shapiro–Wilk test, *P* < 0.05). With this interval, we covered approximately 99.7% of the WT population and we were able to define TF KOs of interest as the ones that fell outside this range. To reduce the outlier effects, we compared the median of the different selected progenies from each cross.

To analyze the circadian defects between the obtained results using the core and the circadian output reporters we applied a *t*-test comparing them, discarding samples that showed different results (*P* < 0.05).

### *N. crassa* knockout complementation

Selected KO strains from the primary genetic screen were complemented by electroporating their conidia with an amplicon containing the corresponding gene, aiming at replacing the *hph* cassette located in the respective knockout loci (Colot *et al.* 2006; Collopy *et al.* 2010). The complementation cassettes were constructed by yeast recombination cloning (Oldenburg *et al.* 1997; Raymond *et al.* 1999) containing 5’- and 3’-Flanking regions with the ORF plus a V5-tag and the *bar* cassette for resistance selection (Collopy *et al.* 2010). Subsequently, the selection was made through microconidiation to obtain homokayotic strains containing the complemented genes at their endogenous loci (Ebbole and Sachs 1990). Thus, complementation was conducted on a subset of particular KOs derived from the screen, to confirm that the absence of a specific TF encoding gene is the cause of the observed period defect. As the absence of some TFs leads to conidial and growth problems (Carrillo *et al.* 2017), we focused instead on strains of interest that could be easily subjected to a transformation protocol. Thus, we complemented KOs for NCU01238, NCU00499, NCU10006, and NCU08999, which were transformed with a cassette reconstituting the missing ORF, plus a V5 tag: complementation of the four above-mentioned mutant loci recovered a WT period phenotype.

## Data availability

Strains and plasmids are available upon request. Circadian data sets associated with the genetic screen are available at https://biodare2.ed.ac.uk/ (Zielinski *et al.* 2014) as “*Neurospora TF Circadian Clock*” and have been also uploaded as supplementary tables to figshare: https://gsajournals.figshare.com/articles/figure/Supplemental_Material_for_Mu_oz-Guzm_n_Caballero_and_Larrondo_2021/14036507?file=26476886.

## Results

### Primary circadian screen

To identify novel regulators impacting the *N. crassa* circadian clock we adopted a reverse genetic strategy, focusing on putative TFs encoded in its genome (Borkovich *et al.* 2004; Tian *et al.* 2011; Weirauch *et al.* 2014). The screen consisted of analyzing the behavior of circadian luciferase reporters in the absence of individual TFs. As indicated in the methods section, this was achieved by crossing strains missing a particular TF, available from the *N. crassa* KO collection, with strains containing a circadian luciferase reporter. Diverse biological and technical issues limited the current extensiveness of this screen: we started our study with a list of 289 genes encoding for putative TFs in the *N. crassa* genome, defined from previous work from our lab (Weirauch *et al.* 2014), adding later on other putative TF encoding genes (Borkovich *et al.* 2004; Tian *et al.* 2011; Carrillo *et al.* 2017), yielding a final list of 302 TF possible candidates summarized in Supplementary Table S1. Nevertheless, KOs for 45 of these genes were unavailable in our version of the *N. crassa* KO collection (Colot *et al.* 2006) when we started the screen (Supplementary Table S1; KOs not available), while some of the genes were essential and therefore obtaining such mutants as homokaryons were not possible (Giaever and Nislow 2014; Carrillo *et al.* 2017). Thus, for the available 257 KOs, sexual crosses were conducted with a strain containing a clock-luciferase reporter, in order to analyze the effect of deleting specific TFs. From this long list, 91 crosses failed to yield enough *hyg^R^*, *luc^+^* offspring for the circadian luciferase analyses.

Genetic linkage issues can explain some of these 91 nonproductive crosses; 41 KO loci are in the same chromosome (LGI) as *his-3*, implying a genetic linkage between the *hph* cassette and the luciferase reporter. Therefore, for these unsuccessful crosses (based on a reporter strain where luciferase was inserted at *his-3*) (Honda and Selker 2009), we conducted new crosses for 18 knockouts, but this time utilizing a *frq-c-box* reporter located at a different region of LGI; the *csr-1* locus (Bardiya and Shiu 2007), which allowed obtaining progeny for 11 additional TF Knockouts, leaving only 80 unsuccessful crosses (Supplementary Table S1; failed offspring) of which 30 can be clearly attributed to genetic linkage. Importantly, among these 80, many of the KOs appeared to be associated with developmental and growth problems (Carrillo *et al.* 2017), partially explaining the failure to obtain successful progenies. Thus, *in toto*, this screen circadianly examined a total of 177 TFs knockouts, corresponding to ∼60% of the cohort of putative *N. crassa* transcriptional regulators (Supplementary Table S1; analyzed strains). The resulting progenies from each cross, and the corresponding WT controls (parental strains and hygromycin sensitive siblings) were monitored for luciferase expression, and analyzed through the Biodare2 platform, focusing on period change (Δτ) (Moore *et al.* 2014; Zielinski *et al.* 2014) and plotted accordingly as shown in Supplementary Figure S1. Progeny of two of the 177 crosses were not included in the plots as they were cataloged as arrhythmic: Δ*wc-1* and Δ*wc-2*, which is expected as they correspond to core clock components (Supplementary Figure S1). Interestingly, the analysis of Δ*NCU02666* first suggested that its absence also abrogated rhythms, as it exhibited extremely low and apparently arrhythmic LUC signals. Nevertheless, normalization of the data revealed weak, but rhythmic oscillations for that reporter (*c-box-luc*), whilst analysis with a different one (*con-10^luc^*, see next section) confirmed that in Δ*NCU02666* the clock still runs with a WT period (Supplementary Figure S2). The reason why in this mutant the behavior of the *c-box* reporter is extremely weak, although the core clock still runs, remains to be determined.

**Figure 1:**
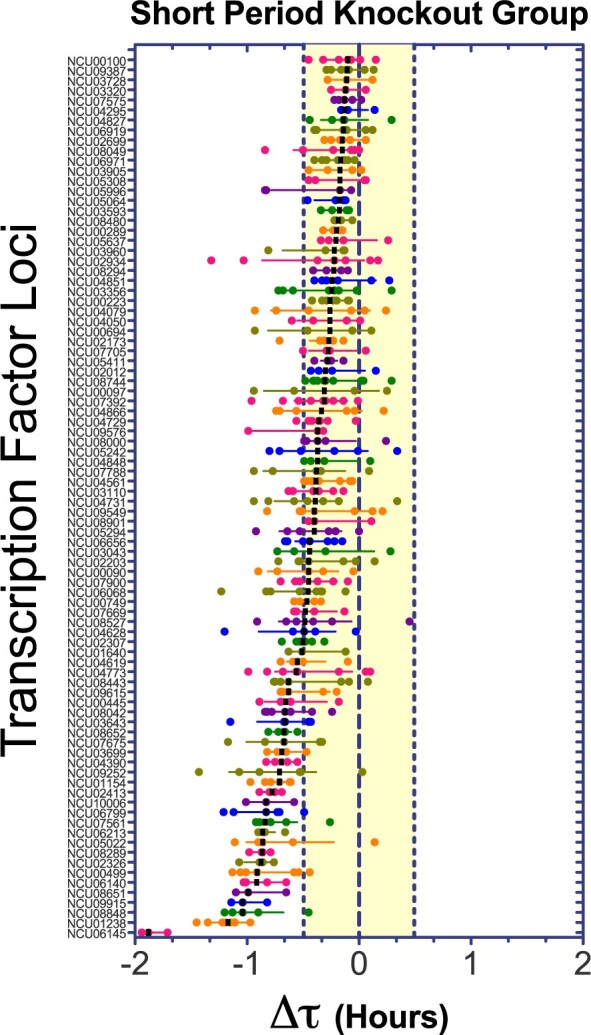
Primary circadian screen of TFs KO strains (short period group). The plot of the short period KO group depicts the **Δτ** values for 88 KOs crosses. Each dot reflects the value for a replicate, the black bar is the median for each KO population and the error bars are the interquartile range. Transcription factor loci are ordered by median values and the yellow filled range reflect the behavior of the 99.7% of the WT population. Twenty-nine strains are outside the range and are selected as TF KO candidates with shorter periods.

**Figure 2:**
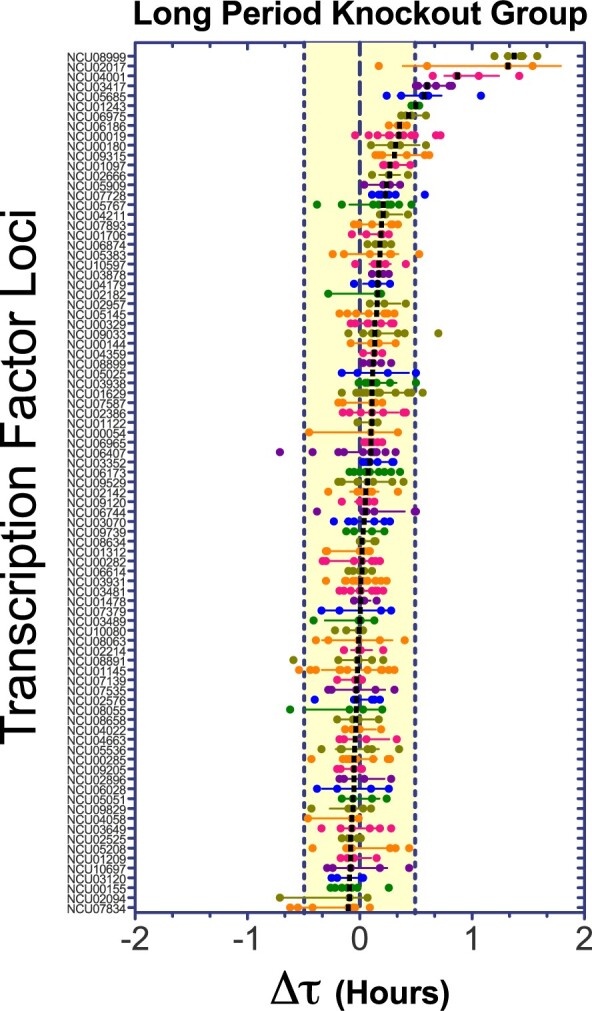
Primary circadian screen of TF KO strains (long period group). The plot of the long period KO group depicts the **Δτ** values for 87 KOs crosses. Each dot reflects the value for a replicate, the black bar is the median for each KO population and the error bars are the interquartile range. The transcription factor loci are ordered by median values, where the yellow filled range reflect the behavior of the 99.7% of the WT population. Based on this, seven KO strains were selected as candidates with a longer period.

To select for the strongest circadian phenotypes in our screen, we defined upper and lower thresholds based on a tolerance interval of three standard deviations of the WT mean values for Δτ (see Materials and Methods; Supplementary Figure S3); this tolerance interval corresponds to ±0.49 hours. With this approach, we covered ∼99.7% of the WT group (assuming a normal distribution, Shapiro–Wilk test *P* < 0.05). The selection of TF KOs of interest comprise strains with calculated median outside this tolerance interval (Zhang *et al.* 2009). For a better visualization of the scattered Δτ data emerging from the plotted 175 crosses, it was separated in two groups: strains exhibiting shorter (Figure 1) and longer (Figure 2) periods. The results of our primary screen analyses are summarized in Supplementary Table S2, indicating the experimental identifiers, calculated period change (Δτ), number of biological replicates, and the descriptive statistics for each of the studied TF KOs; also, we listed in a secondary supplementary table (Supplementary Table S3), the raw results from each individual strain retrieved from the BioDare2 platform for simpler and faster access.

Out of this analysis, only 36 KOs showed statistically significant differences in the primary screen, based on the above-mentioned criteria of three standard deviation from the wild-type population mean (Zhang *et al.* 2009). Notably, 30 out of 36 TF KOs displayed shorter period, results that contrast with what has been observed in similar screens conducted in other circadian models, where most of the identified genes affecting the clock yield longer periods (Matsumoto *et al.* 2007; Hirota *et al.* 2008; Zhang *et al.* 2009; Agrawal and Hardin 2016). To ensure that the circadian change in these 36 candidates was caused by the removal of the specific locus of interest, we also analyzed WT siblings for each selected KO. This additional evaluation helped reducing the deviation associated with technical noise derived from the analysis of different plates and/or camera runs, or for potential unlinked spontaneous mutations present in the KO or emerging during the sexual crosses (Watters and Stadler 1995).

### Confirmation of circadian phenotype by output reporters

To confirm that the clock defects observed in our screen with a minimal core clock reporter (*c-box*-*luc*) were actually due to circadian alterations, and not to unidentified technical issues, such as low reporter expression or other reasons (see Supplementary Figure S2), we evaluated the behavior of a different reporter in the selected TF KOs. For this, we utilized the output gene *con-10* (NCU07325), which exhibits robust circadian expression (Lauter and Yanofsky 1993; Hurley *et al.* 2014). *con-10* is a vastly studied gene, expressed in late stages of conidial differentiation (Roberts *et al.* 1988; Olmedo *et al.* 2010a), responds to light (Olmedo *et al.* 2010b; Wu *et al.* 2014), and is highly expressed during carbon starvation, similar to our experimental conditions, (Xiong *et al.* 2014, 2017) among others regulations (Kays *et al.* 2000; Thompson *et al.* 2008; Wang *et al.* 2012; Pengkit *et al.* 2016; Dekhang *et al.* 2017). We created a *con-10^luc^* reporter by integrating *luc* at the corresponding locus obtaining a fusion between the latter and the *con-10* ORF (Olivares-Yanez *et al.* 2016).

**Figure 3:**
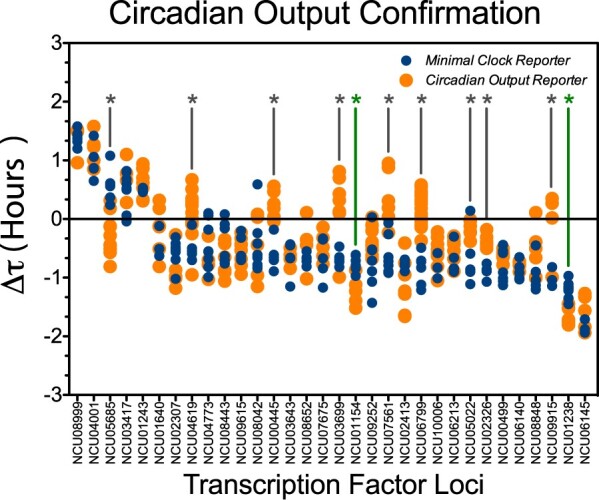
Confirmation of TF candidates using a Circadian Output Reporter. Thirty-two TF candidates were successfully analyzed utilizing the circadian output reporter *con-10^luc^* (orange dots) and *frq_c-box_-luc* (blue dots), where each dot is a biological replicate. Eight candidates were statistically different between both reporters (gray asterisk), showing a **Δτ** value for the circadian output reporters close to zero. Two strains (*Δsub-1* and *ada-9*) also showed significant differences between both reporters (green asterisk), but with a shorter period for *con-10^luc^* compared to *frq_c-box_-luc*. All things considered, the intersection of both screens allowed identifying twenty-three TF as significantly impacting circadian period in Neurospora.

This secondary analysis (Figure 3) reduced the number of TFs of interest derived from the primary screen. Four of the 36 candidate TFs were disregarded in the following analysis for experimental issues; three of them (NCU04390, NCU08289, and NCU08651) failed to produce successful offspring with the *con-10^luc^* reporter, whereas the KO for NCU02017 severely affected the expression of the circadian output reporter; leaving further confirmation of these KOs pending. Nine strains were also left out of the refined list of TFs of interest as in the *con-10^luc^* analyses since period differences did not statistically recapitulate what had been observed with the core clock reporter (Figure 3).

Thus, interestingly, the use of two different circadian reporters allowed us to observe differences in the degree of circadian alteration of several TF KOs strains (Supplementary Figure S2). These differences could relate to a direct effect of the candidate TF in both pathways: alterations on the *N. crassa* clock and in output pathways (*con-10*), versus a major effect only in the former and compensatory mechanisms in the latter (albeit this would be less likely). A clear example of a KO impacting both core clock function and the output pathways while severely compromising the quality of *con-10* rhythms is Δ*ada-*2 (*NCU02017*), where the expression of *con-10^luc^* appears severely affected. This TF KO shows defects in both sexual and asexual development (Sun *et al.* 2019) and differential gene expression upon carbon source differences (Reilly *et al.* 2015), conditions where *con-10* regulation is affected (Wang *et al.* 2012; Xiong *et al.* 2014, 2017).

In terms of period, Δ*sub-1* (NCU01154) and Δ*ada-9* (NCU01238), where significantly shorter when examining output compared to the core clock reporter (Figure 3). Both of these TFs had been previously associated with *con-10* regulation: in Δ*sub-1*, light induction of *con-10* is reduced (Sancar *et al.* 2015a), and in the case of ADA-9, this TF is capable of interacting with RCO-1 (Olivares-Yanez *et al.* 2016), a transcriptional co-repressor that drastically affects *con-10* expression (Yamashiro *et al.* 1996). In addition, both KOs show alterations on sexual and asexual development (Carrillo *et al.* 2017), conditions where *con-10* expression is differentially regulated, as commented above. Yet, it is not obvious to explain that period for a particular KO would yield so different results with the core-clock and output reporters.

Thus, applying a conservative criterion restricting the list to those which absence showed significant period changes in both screens, we identified 23 TF encoding genes, which corresponds to ∼7.6% of the total number of putative TFs in *N. crassa* (and a ∼13% of the successfully analyzed for *luc* expression). Importantly, other clock screens based on similar reverse genetics approaches have shown variable results. For example, while in a broad screen using human cells the rate of genes of interest was near 1% for a total of 22,468 genes, or a smaller subgroup (Maier *et al.* 2009; Zhang *et al.* 2009), depending on the size of the screen or the category of the analyzed genes such rates can go up. Thus, genes flagged as of interest were ∼3.8% of 133 circadianly expressed genes screened in Drosophila (Matsumoto *et al.* 2007), compared to ∼22% of 86 phosphatase encoding genes in the same organism (Agrawal and Hardin 2016). To our knowledge, there are no published studies exclusively focusing on the circadian impact of TFs, although TFs with a clock-related function have been already identified in unbiased screens (Matsumoto *et al.* 2007).

### The TFs of interest are associated with multiple processes

Thus, based on the strict criteria of showing period alterations when assessing with both reporters, we have identified 23 TFs with strong and reproducible circadian defects, which appear to be involved in a broad range of cellular processes, as it is indicated in the next paragraphs for each one. The absence of the corresponding ORFs in the progeny of these 23 KOs was confirmed by PCR, and for four particular KOs (that will be further pursued for mechanistic studies) we conducted complementation assays (see below and Supplementary Table S4).

**Figure 4:**
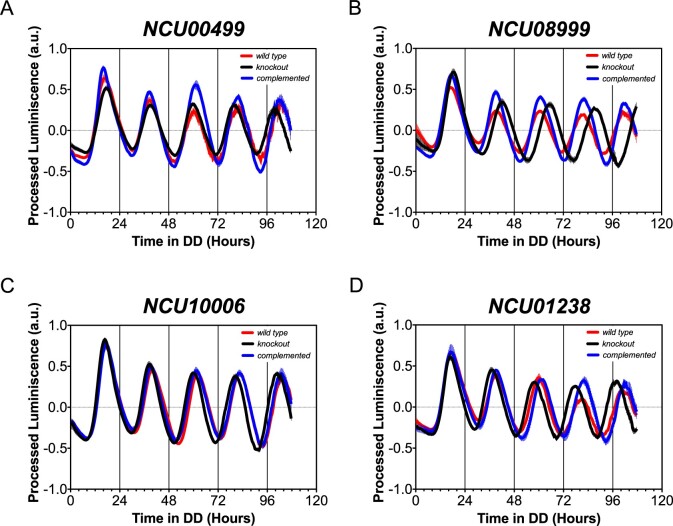
Validation of TFs of interest by complementation. Four KO strains from the selected candidates were individually transformed, as explained in methods, with a construct that allowed reinserting the missing TF locus. KOs for the TF encoding genes: A, *ada-1* (*NCU00499*); B, *cbf-1* (*NCU08999*); C, *sgr-30* (*NCU10006*), and D, *ada-9* (*NCU01238*) were complemented. The red lines, depict the oscillations of the *wild type*s; black, the traces of the KO strains, whereas the blue lines, correspond to the complemented KO strains. For each strain, we used at least three biological replicates. All complementations successfully restored the wild-type circadian phenotype.

***NCU08999*:** (Δτ = +1.38 h) this locus encodes for a bHLH TF, which is an ortholog of the yeast *centromere binding factor 1* (*cbf-1*) (Stoyan *et al.* 2001). This gene is known to be expressed late after phytosphingosine treatment (Videira *et al.* 2009). Recently this *N. crassa* TF was described to have a similar circadian phenotype (∼2 h lengthened period), as determined by race tube assays (Cao *et al.* 2018). Interestingly, the authors failed to observe circadian expression of luciferase in their characterization of this mutant, which could be partially explained by their experimental settings (see Discussion), a discrepancy we have seen with other mutants like Δ*rco-1* (Zhou *et al.* 2013; Olivares-Yanez *et al.* 2016). The period phenotype associated with Δ*NCU08999* was restored to WT upon complementation (Figure 4B).

***NCU04001*:** (Δτ = +0.87 h) identified as *female fertility 7* (*ff-7*), this zinc-finger TF was found to interact with SUB-1 (NCU01154) in regulating the expression of several genes in LL and DD conditions. This transient complex is able to interact with WCC upon light exposure, modulating several genes (Sancar *et al.* 2015a). ADV-1 (NCU07392) regulates *NCU04001/ff-7* expression in a light dependent manner (Dekhang *et al.* 2017). *ff-7* is subjected to metabolic regulation and its expression is decreased in a Δ*col-26* (NCU07788) strain in amylose (Xiong *et al.* 2017), and it is also reduced in a strain overexpressing CSP-1 (NCU02713) in light conditions (Sancar *et al.* 2011).

***NCU03417:*** (Δτ = +0.52 h) encodes for a hypothetical C6 zinc-finger containing protein. Its expression in DD is altered in the absence of MAK-1 (Mitogen-Activated Protein Kinase) (Bennett *et al.* 2013).

***NCU01243:*** (Δτ = +0.50 h) corresponds to *zinc-finger 41* (*znf-41*), a TF highly conserved in many pathogenic fungi. In *N. crassa*, it is induced by menadione, and its absence leads to enhanced ROS sensitivity (Zhu *et al.* 2013). It is a light-responsive gene, directly regulated by WCC (Smith *et al.* 2010), while also displaying rhythmic expression (Hurley *et al.* 2014).

***NCU01640:*** (Δτ = −0.51 h) corresponds to a C2H2 TF named *regulatory particle non-ATPase-like-4* (*rpn-4*). *rpn-4* is a light-responsive gene, whose expression is up-regulated by ADV-1 (Dekhang *et al.* 2017) and down-regulated by CSP-1 (Sancar *et al.* 2011). Both of these TFs are rhythmically expressed (Hurley *et al.* 2014; Dekhang *et al.* 2017), and are related to responses to particular sugar availability, implying a possible metabolic control over *rpn-4*. Thus, expression of the latter and of *adv-1*, are decreased in the presence of amylose in a Δ*col-26* strain (Xiong *et al.* 2017). Also, the expression of this gene is strongly activated under conditions which challenge cell integrity; such as phytosphingosine treatment, an inducer of programed cell death in *N. crassa*, (Videira *et al.* 2009), and conditions that favor cell fusion and cell-to-cell communication, regulated by ADV-1 and PP-1 (NCU00340) (Fischer *et al.* 2018). This gene exhibits rhythmic expression (Hurley *et al.* 2014).

***NCU02307*:** (Δτ = −0.51 h) is a light-responsive gene encoding for a zinc-finger TFs, up-regulated indirectly by ADV-1 (Dekhang *et al.* 2017), with reduced expression in a *csp-1* overexpressing strain (Sancar *et al.* 2011). It varies differentially between new and old vegetative tissue (Tian *et al.* 2011), and it is mostly expressed in aerial hyphae compared to mycelia (Greenwald *et al.* 2010). Albeit this TF has not been well characterized yet, its expression is strongly tied to plant cell-wall degradation, being highly expressed under such conditions (Tian *et al.* 2009), depending on XLR-1, a well-known TF involved in hemicellulose degradation (Sun *et al.* 2012a). Its expression is also differentially regulated in starch, where it is repressed by the TF COL-26 (Xiong *et al.* 2017). *NCU02307* has been reported as exhibiting rhythmic expression, with a peak in the evening (Hurley *et al.* 2014).

***NCU04773*:** (Δτ = −0.56 h) encodes for a conserved fungal hypothetical protein containing a copper ion-binding domain. A related ortholog is GRISEA, a copper-dependent TF in *Podospora anserina* or yeast MAC1 (Borghouts and Osiewacz 1998). Podospora lacking GRISEA shows an increased lifespan by reduction of ROS and ATP, this through switching from a copper-dependent to an iron-dependent respiration system (Borghouts *et al.* 1997; 2001; Gredilla *et al.* 2006).

***NCU08443*:** (Δτ = −0.63 h) This gene encodes for a zinc ion-binding TF. It has been shown to display rhythmic expression by RNA-Seq, with a morning peak of expression (Hurley *et al.* 2014).

***NCU09615*:** (Δτ = −0.63 h) encodes for a zinc TF named *vegetative asexual development 14* (*vad-14*) (Carrillo *et al.* 2017) and is a light-responsive gene, with a late-light expression pattern—between 60 and 120 min—(Wu *et al.* 2014), directly regulated by WCC (Smith *et al.* 2010) and indirectly by ADV-1 (Dekhang *et al.* 2017). It has been described to have rhythmic expression, with a morning peak (Hurley *et al.* 2014). Also, it is induced by starch-related carbon sources and not by simple sugars such as maltose (Xiong *et al.* 2017). In cell viability assays its levels are increased (Hutchison *et al.* 2009).

***NCU08042*:** (Δτ = −0.64 h) this locus encodes for the C6 zinc-finger TF *cellulose degradation regulator 2* (CLR-2). Identified by its severe growth defect on crystalized cellulose (Avicel) (Coradetti *et al.* 2012). CLR-2 regulates the expression of several cellulolytic genes (Coradetti *et al.* 2013), and itself is induced by cellulose in a CLR-1 (NCU07705) dependent manner (Coradetti *et al.* 2012; Znameroski *et al.* 2012; Xiong *et al.* 2017).

***NCU03643:*** (Δτ = −0.66 h) Encodes for a zinc-finger TF ortholog of *cutinase transcription factor 1 beta* (*ctf1β*) (Li *et al.* 2002; Tang *et al.* 2011). It is a light-responsive gene (Dong *et al.* 2008; Chen *et al.* 2009) whose expression appears to be regulated by FF-7 (Sancar *et al.* 2015a), and it is also affected by menadione (Zhu *et al.* 2013). It is downregulated in the absence of *col-26*, under amylose conditions (Xiong *et al.* 2017), and it exhibits a delayed up-regulation to quinic acid (QA), likely mediated by QA-1F (NCU06028), the main controller of the QA cluster in *N. crassa* (Patel and Giles 1985; Tang *et al.* 2011). It is positively regulated by direct binding of ADV-1, providing its basal expression (Dekhang et al. 2017). CTF1β has been predicted as an “activator” of the delayed group of metabolic genes up-regulated after QA addition (Tang *et al.* 2011), and it has also been reported as exhibiting rhythmic expression (Hurley *et al.* 2014).

***NCU08652*:** (Δτ = −0.67 h) this locus encodes for hypothetical C6 zinc-finger TF, described based on its knockout phenotype as *slower growth rate 31* (*sgr-31*) (Carrillo *et al.* 2017). It has a differential expression when grown under maltose compared with other carbon source or sucrose (Xiong et al. 2017).

***NCU07675*:** (Δτ = −0.67 h) Encodes for a C6 zinc-finger TF defined as *tall aerial hyphae 4* (*tah-10*). Its expression is affected by amylose, showing decreased levels in the absence of *col-26* (Xiong *et al.* 2017), with high expression in asexual reproduction conditions (Wang *et al.* 2019). Its expression is reduced in a CSP-1 overexpression strain, albeit it is not a direct target of this TF (Sancar *et al.* 2011).

***NCU01154*:** (Δτ = −0.71 h) s*ubmerged protoperithecia 1* (*sub-1*), a GATA TF, is an early light-responsive gene, directly regulated by WCC (Chen *et al.* 2010; Smith *et al.* 2010; Wu *et al.* 2014), exhibiting a rhythmic expression with a morning pattern (Hurley *et al.* 2014). Functionally, it is able to dynamically interact with FF-7, and regulate the expression of several genes in light and darkness, also acting synergistically with WCC in light-responsive genes (Sancar *et al.* 2015a), connecting light responses and fungal development (Kasuga *et al.* 2005; Wang *et al.* 2019). It is differentially expressed in young versus old tissue (Tian *et al.* 2011), favored in sexual reproduction and down-regulated in asexual development (Wang *et al.* 2019). It is also subjected to metabolic regulation under different carbon sources, such as maltose and amylose in a *col-26* dependent manner (Xiong *et al.* 2017).

***NCU09252*:** (Δτ = −0.71 h) Encodes for a hypothetical C2H2 TF whose expression in darkness depends on SUB-1 and FF-7 at basal levels (Sancar *et al.* 2015a), as well as on the TF *vad-5* (NCU06799) (Sun *et al.* 2012b). It has been described as clock-controlled and temperature-regulated (Nowrousian *et al.* 2003), and is member of the over-expressed genes of the starch-regulon in *N. crassa*, dependent on COL-26 (Xiong *et al.* 2017), and preferentially expressed in young versus old tissue (Tian *et al.* 2011).

***NCU02413*:** (Δτ = −0.78 h) defined as *response regulator 2* (*rrg-2*), is part of a two-component regulatory system for stress response in *N. crassa* (Catlett *et al.* 2003; Froehlich *et al.* 2005; Jones *et al.* 2007); it has been described as containing a truncated HSF DNA-binding domain and it is involved in ROS responses (Banno *et al.* 2007; Thompson *et al.* 2008). In addition, it exhibits a repressive effect on the secretion of lignocellulases, based on its requirement in the ER stress response (Fan *et al.* 2015). It is also downregulated during cell viability assays (Hutchison *et al.* 2009), being more expressed in mycelia than in aerial hyphae (Greenwald *et al.* 2010).

***NCU10006*:** (Δτ = −0.83 h) Named as *slow growth rate 30* (*sgr-30*) (Carrillo *et al.* 2017), is a gene coding for a C2H2 TF with an increased expression under high level of phosphate in the media (Gras *et al.* 2013), with no additional function or process associated with this gene. Complementation of the mutant back with the *NCU10006* gene recovered WT period (Figure 4C).

***NCU06213*:** (Δτ = −0.85 h) *zinc-finger transcription factor 9* (*znf-9*). This gene has shown to display rhythmic expression, with an evening peak (Hurley *et al.* 2014).

***NCU00499*:** (Δτ = −0.91 h) corresponds to *all development altered 1* (*ada-1*), a bZIP, of which KO yields a strong growth phenotype (Carrillo *et al.* 2017). This TF has an increased expression in young versus old tissue, and mostly in aerial hyphae (Greenwald *et al.* 2010; Tian *et al.* 2011), and displays rhythmic expression (Hurley *et al.* 2014). The period phenotype observed in Δ*ada-1* was reverted by complementation (Figure 4A).

***NCU06140*:** (Δτ = −0.92 h) *vegetative and sexual development* (*vsd-8*) (Carrillo *et al.* 2017) encodes for a MYB TF. Under light exposure, it is up-regulated by SUB-1 and modulated by ADV-1 (Sancar *et al.* 2015a; Dekhang *et al.* 2017); in darkness, its basal levels are diminished in the absence of *ff-7* and *sub-1*, being also a direct target of FF-7 (Sancar *et al.* 2015a). It exhibits metabolic regulation, having an increased expression under amylose in a Δ*col-26* strain (Xiong *et al.* 2017), and is a downstream target of the MAP kinase signaling through the regulation by the TF PP-1 (Gras *et al.* 2013; Leeder *et al.* 2013).

***NCU08848*:** (Δτ = −1.04 h) is a hypothetical protein with a zinc-ion binding domain. Its expression varies during conidial germination (Kasuga *et al.* 2005).

***NCU01238*:** (Δτ = −1.17 h) is a PHD TF named as *all developmental alteration 9* (*ada-9*). It is capable of interacting with the co-repressor RCO-1, a transcriptional regulator, devoid of a DNA binding domain, known to impact clock regulation and which absence leads to lengthened period (Olivares-Yanez *et al.* 2016). Complementation of Δ*NCU01238* recovered WT period (Figure 4D).

***NCU06145*:** (Δτ = −1.88 h) Encodes for a C2H2 TF named as *really interesting gene 6* (*ring-6*). It is also a light down-regulated gene, bound by ADV-1 (Dekhang *et al.* 2017), with an expression that is affected by the presence of maltose (Xiong *et al.* 2017). This KO strain does not show the characteristic displays of apical branching when is exposed to cold shock (Watters *et al.* 2018). In our hands, Δ*NCU06145* yielded the shortest period among the mutants identified in this screen.

## Discussion

Our study constitutes, so far, one of the most extensive reverse genetic analyses concentrating on the *N. crassa* clock utilizing luciferase as a proxy for circadian molecular phenotypes. Such an approach is a major advance compared to previous *N. crassa* circadian forward genetic screens based on race tubes (Feldman and Hoyle 1973; Feldman and Atkinson 1978), due its fine spatiotemporal resolution, ideal for measuring key clock parameters as period, and facilitating high-throughput analyses by monitoring multiple strains and replicas simultaneously (Gooch *et al.* 2008; Larrondo *et al.* 2012; Cha *et al.* 2015). Undeniably, the race tube assay is a robust method for circadian screenings, which led to the identification of *frequency* (Feldman and Hoyle 1973; Loros and Feldman 1986; Aronson *et al.* 1994a) and several other clock affecting loci (Lakin-Thomas *et al.* 1990; Loros and Dunlap 2001; Morgan *et al.* 2001; Lakin-Thomas et al. 2011), some of which were subsequently functionally characterized such as *prd-1*, *prd-4*, and *prd-6* (Pregueiro *et al.* 2006; Emerson *et al.* 2015; Adhvaryu *et al.* 2016). However, race tubes may overlook mutations of interest, as any alterations which impact conidiation *per se* would show overt arrhythmicity, although core circadian function may remain intact, or even with distorted parameters. Indeed, KOs of TFs associated with asexual growth (Colot *et al.* 2006) could yield confusing or inconclusive results on race tube assays, as conidiation banding would be obscured (Baker *et al.* 2012).

Out of all the screened TFs (∼60% of 302 in *N. crassa*), WC-1 and WC-2 continue to be the only ones essential for the clockworks. Nevertheless, extensive analysis of the remaining TFs is needed in order to identify whether another regulator plays a critical role, most likely commanding the expression of clock components other than FRQ, such as the WCC, CK1, or FRH encoding genes. It is noteworthy, though, that none of the screened TF causes major clock alterations and that, although a limited number of TFs affect period, they do so within a rather narrow range of hours. The low number of TFs strongly impacting the clockworks could be explained by two possibilities: a “*TF”* or a “*circadian system”* interpretation. The former is the most parsimonious interpretation and implies that the circadian core oscillator is not critically regulated by a significant number of TFs, as opposed to what occurs in other regulatory systems. On the other hand, we have the *“circadian system* explanation,” which argues of a pervasive robustness of circadian systems, taking the clock phenotype as a robust structure resilient to individual genetic perturbations (Kitano 2002; Félix and Barkoulas 2015). A corollary of the latter premise is that some genetic perturbations may require a particular environmental stimulus, or combination of stimuli, to reveal an important role in the clockworks (Kitano 2002; Félix and Barkoulas 2015), this due the dynamic reprogramming of regulatory networks, which enables cells to modify network topology to adapt to complex environmental perturbations (Hu *et al.* 2016; Swift and Coruzzi 2017). Thus, a given regulator may not be identified as important in metabolic or temperature compensation unless defined sugar levels or temperatures are tested (Sancar *et al.* 2012; Olivares-Yanez *et al.* 2016). Indeed, supporting this idea are additional pieces of evidence to discuss. For example, CSP-1 (NCU02713), a TF implicated in metabolic compensation of the clock (Sancar *et al.* 2011, 2012), has been shown to modulate circadian period only under high glucose conditions, since under low glucose levels, no circadian alterations are appreciated (as we saw herein for Δ*csp-1* in our screen conditions). Likewise, we have also reported that Δ*rco-1* is a mutant which exhibits a longer period, but is shortened to almost WT levels as glucose concentration is increased (Olivares-Yanez *et al.* 2016). Herein, we were able to observe clear luciferase rhythms in Δ*cbf-1*, contrary to what was reported for this mutant (Cao *et al.* 2018); such difference could be due to the culture conditions used by the authors to monitor luciferase (GAI *et al.* 2017), which are similar to ours, except that theirs (FGS, consisting of 0.05% fructose, 0.05% glucose and 2% sorbose), contains other sugars and promotes colonial growth (ours is only 0.03% glucose). Importantly, the molecular rhythms we detected by bioluminescence confirmed the increase in period, which was described for this mutant based on race tube assays. Indeed, by parsimony it is expected that a robust overt conidiation rhythm, as seen for Δ*cbf-1* (Cao *et al.* 2018), should be accompanied by molecular rhythms in *luc* expression, as we have reported herein. Importantly, these case studies exemplify the advantage of having a minimal clock promoter reporter (*frq_c-box_-luc*) that can be easily crossed to mutants of interest, with no concerns for ripping as it would occur for full *frq* promoter-reporters (Gooch *et al.* 2008). Likewise, the *con-10^luc^* reporter, which we also utilized in this screen, can be easily crossed as it corresponds to a *luc* knock-in at the *con-10* locus, creating a translational fusion reporter (Olivares-Yanez *et al.* 2016). Such resources are extremely useful considering the existing *N. crassa* KO collection (Colot *et al.* 2006), as well as the great assortment of historical mutants at the FGSC (McCluskey *et al.* 2011), which may be hard to screen by the conventional race tube assay, since many exhibit growth problems.

Interestingly, most of the 23 TFs candidates fall in two well-studied biological processes in *N. crassa*: light responses and carbon availability (Supplementary Table S4). Light triggers well-defined transcriptional responses in this fungus, mediated by the photoreceptor and TF WC-1, being also one of the main inputs to the clock, able to reset and entrain it (Montenegro-Montero *et al.* 2015). Several of the 23 TFs of interest appear to be subjected to both light- and circadian-regulation. Starting from WCC direct targets, we observed the case of *sub-1*, *znf-41*, and *vad-14*, where the three of them have been both reported to be clock-controlled and light-responsive genes, probably members of the second-tier of TFs able to control downstream circadian effectors (Smith *et al.* 2010; Hurley *et al.* 2014). Based on previous works (Smith *et al.* 2010; Sancar *et al.* 2011; Dekhang *et al.* 2017), we know that *adv-1* and *csp-1* are members of this second-tier, implying a plausible role on the clock-controlled and light-response of *rpn-4*, *ctf1β*, and *NCU02307* genes, being these last three part of a third-tier of transcriptional regulation which could include a transcriptional feedback to the clock (Sancar *et al.* 2011; Dekhang *et al.* 2017). From the 23 TFs of interest, three of them have been described as having circadian expression, but no regulation by light: *znf-9*, *ada-1*, and *NCU09252* (Hurley *et al.* 2014), being the latter the only known connected to SUB-1, a second-tier TF (Sancar *et al.* 2015a). The TFs of interest *ring-6*, *vsd-8*, and *ff-7*, have not been reported to have circadian expression, but are light-responsive genes regulated by ADV-1 (Dekhang *et al.* 2017). It is outstanding that out of the 23 TFs we identified as modulating period, 9 appear to be under clock-regulation themselves, intertwining aspects of output control back to core clock dynamics. Previous studies (Hurley *et al.* 2014; Sancar *et al.* 2015b), have indicated that ∼10% of the *N. crassa* TF genes are circadianly expressed; our results provide additional hints unveiling potential rhythmic transcriptional networks around the *N. crassa* circadian clock (Zhang and Kay 2010). Thus, an overrepresentation of the TFs of interest which are not only rhythmic but also, through yet unclear mechanisms, feedback to modulate circadian period, blurring the already fine line and directionality between clock output and core mechanisms. Moreover, we predict that by altering the growth conditions (*i.e.*, varying nitrogen/sugar source or content) several of the clock phenotypes observed for a given set of TFs will change, potentially uncovering new aspects of the intricate mechanisms undelaying metabolic compensation, in the case of CSP-1 (see above) ( Sancar *et al.* 2011; 2012), or in developmental control, exemplified by SUB-1 and its role in the asexual-sexual switch (Wang *et al.* 2014, 2018).

*N. crassa* is a fungus that can be found growing on different type of plant tissues, and therefore, sensing nutrient availability is one a key aspect of its biology, involving a series of fast and accurate responses upon substrate recognition (Horta *et al.* 2019). Two of the genes of interest, *clr-2* and *NCU02307* are highly expressed upon exposure to complex substrates, like cellulose and hemicellulose respectively, each of them under the control of the main TFs regulating the expression on these substrates, CLR-1 and XLR-1 (Tian *et al.* 2009; Sun *et al.* 2012a). CLR-2 is a TF involved in the decomposition of the plant cell wall, but mainly controlling the expression of cellulases, and having no connection to the circadian clock components in cellulose conditions (Coradetti *et al.* 2012; Znameroski *et al.* 2012), whereas recent studies have shown how the clock nicely functions when *N. crassa* grows on plant material (Diaz and Larrondo 2020). Regarding complex carbon sources, for starch there is a defined starch-regulon commanded by the TF COL-26, with highly expressed genes under amylose conditions, where we can find the candidate gene *NCU09252*. Not part of the starch-regulon, but regulated by COL-26, are *ff-7*, *rpn-4*, *sub-1*, *ctf1β*, *tah-10*, and *vsd-8*, whereas *vad-14* responds to amylose but no depending on COL-26 (Xiong *et al.* 2017). Simpler carbon source like maltose or glucose show effects in the expression of some of the genes of interest: for maltose there is an increase in the levels of *ring-6*, *srg-31*, and *sub-1* (Xiong *et al.* 2017). Related to glucose metabolic compensation of the *N. crassa* clock, both the co-repressor RCO-1 and the transcriptional repressor CSP-1 have been implicated (Sancar *et al.* 2012; Olivares-Yanez *et al.* 2016), appearing to modulate an ancillary feedback loop to the clock under high glucose conditions. While these candidate genes are not glucose regulated, they are regulated by CSP-1 in response to light, like *rpn-4* and *NCU02307*, the latter a plausible connection between light and metabolic regulation (Sancar *et al.* 2011).

## Conclusions

Our screen, focused on TFs, and utilizing two different clock-reporters, has allowed identification of 23 TFs which absence leads to discrete period changes, most of them associated with period shortening. Although the underlying mechanisms of these circadian phenotypes still remain obscure, it is noteworthy that several of the TFs of interest are, themselves, light- or-clock regulated.

On the other hand, this screen has allowed confirmation of period defects for recently characterized mutants such as Δ*cbf-1*, providing clear evidence of molecular rhythms in TF KO strains where they had been hard to observe using luciferase.

Finally, during this study, we have generated a Δ*TF frq_cbox_-luc* collection, spanning 177 different TFs, which constitute a valuable resource that will expedite new genetic screens contemplating particular single and combined environmental perturbations such as nutrients, pH, and temperature. This will help further unveiling the existence of a dynamic transcriptional network supporting robust and compensated clock function.
